# Racial capitalism and the US formula shortage: A policy analysis of the formula industry as a neocolonial system

**DOI:** 10.3389/fsoc.2022.961200

**Published:** 2022-10-26

**Authors:** Cecília Tomori, Aunchalee E. L. Palmquist

**Affiliations:** ^1^Johns Hopkins University School of Nursing and the Johns Hopkins Bloomberg School of Public Health, Johns Hopkins University, Baltimore, MD, United States; ^2^Department of Maternal and Child Health, Gillings School of Global Public Health, Carolina Global Breastfeeding Institute, University of North Carolina at Chapel Hill, Chapel Hill, NC, United States; ^3^Carolina Population Center, University of North Carolina at Chapel Hill, Chapel Hill, NC, United States

**Keywords:** racial capitalism, colonialism, neocolonialism, breastfeeding, formula industry, multinational corporations (MNC), infant feeding policy, infant and young child feeding in emergencies (IYCF-E)

## Abstract

The U.S. is currently experiencing a formula shortage and an infant feeding crisis that began with a formula recall and the hospitalization of 4 infants, 2 of whom died. Since 1981, governments around the world have been calling for an end to blatant human rights violations made by the commercial milk formula (CMF) industry. These practices not only involve targeting nutritionally vulnerable populations of mothers and newborns to turn a profit, but also actively undermining the implementation of policies, legislation, and regulatory oversight that might compromise their accumulation of wealth. In this paper we analyze the 2022 formula-shortage-as-infant-feeding-crisis through the lens of the history of colonialism and critical theory in the anthropology of reproduction. First, we provide an overview of the colonial roots of the formula industry from a global perspective. We then focus on how the mechanisms of racial exploitation remain entrenched in the U.S. approach to infant feeding policies, regulation and investment, setting the stage for the current infant feeding crisis. Through our analysis of the 2022 infant feeding crisis we demonstrate how the multinational CMF industry perpetuates racial capitalism and racialized health inequities and disparities through its operations as a neocolonial enterprise. Finally, we offer policy interventions and potential solutions that are grounded in structural interventions for more equitable, anticolonial, antiracist infant feeding systems.

## Introduction

On February 17, 2022 a commercial milk formula (CMF) recall at an Abbott Nutrition manufacturing facility in Sturgis, MI set off a cascade of events resulting in a critical CMF shortage and infant feeding crisis across the United States. The US Food and Drug Administration (FDA) and the US Centers for Disease Control and Prevention, along with state and local partners, began investigating consumer complaints or reports to the FDA starting in September 2021 (U.S. Food and Drug Administration, 2022a). Four infants from three states (Minnesota, Ohio, and Texas) were hospitalized with bacterial infection after consuming powdered infant formula (PIF) from the Sturgis plant, and two of them died. Abbott Nutrition voluntarily recalled selected lots of their PIF over potential *Cronobacter sakasakii* and *Salmonella* Newport contamination (U.S. Food and Drug Administration, 2022b). The recalls included both national and international distributions. While Abbott Nutrition denies that their products have evidence of contamination linking them to these four cases, an FDA report notes that “the processes, procedures, and conditions that the FDA observed during its inspection of the Sturgis MI production facility from January 31 to March 18 2022, raise concerns that the powdered infant formula produced at this facility prior to the FDAs inspection carry a risk of contamination” (U.S. Food and Drug Administration, 2022a).

While the quality and safety of PIF raised the public's concern, it was a shortage of PIF that generated shockwaves of panic across the country. Simultaneously, the media coverage highlighted families' stories of hardship, especially among parents whose infants relied on specialized formula products for nutrition. News reports also fueled social controversies surrounding breastfeeding, which effectively drew the public's attention away from the fundamental causes of the formula shortage and inequities in infant feeding safety rooted in systemic racism and structural violence.

Within weeks of the recall and critical shortages of PIF, the FDA, the U.S Department of Agriculture (USDA), and CDC, and several health professional organizations, including the American Academy of Pediatrics (AAP) (American Academy of Pediatrics, 2022a), the Academy of Breastfeeding Medicine (Academy of Breastfeeding Medicine, 2022), and the United States Lactation Consultants Association (USLCA, 2022), released statements to guide families trying to find formula and to assist health care providers to support families in need. At least nine bills have been recently introduced to Congress to address the crisis and attempt to redress systemic failures of policy and the CMF industry. None of them offer any substantive policy or systems interventions to simultaneously support breastfeeding.

On May 19, 2022, President Biden authorized the Defense Production Act to expedite the importation of CMF manufactured outside of the US and relieve domestic CMF shortages (The White House, 2022a). “Operation Fly Formula” facilitated expedited delivery of 70,000 lbs. of Nestlé S.A. PIF from Switzerland to Indiana using U.S. Department of Defense aircraft and personnel. This delivery was the first of multiple planned shipments of both PIF and liquid CMF that were procured with government funding and delivered using military assistance (The White House, 2022b). Meanwhile, Abbott Nutrition's Sturgis, MI facility was authorized to produce a limited supply of specialty and metabolic formulas under strict FDA oversight.

Thus far, the 2022 CMF shortage has been unprecedented in its treatment as a national food security issue warranting military intervention and in the amplification of gaping policy gaps for infant feeding in emergencies. News coverage and social media conversations have tended to dwell on social controversies about “breastfeeding vs. formula feeding,” even though most people who deliver an infant in the U.S. will initiate breastfeeding and then also combine human milk feeding with CMF feeding at some time during an infant's first year of life (Centers for Disease Control and Prevention, 2020). The individualist attention placed on select parents' responses to the crisis and their infant feeding practices without adequate context has shifted public attention away from the fundamental, structural causes of the infant feeding crisis. This crisis is not novel, and its consequences are not equally borne; instead, it is rooted in a deeper history of unethical manufacturing and marketing practices, political lobbying, and profit extraction built on a colonial system of racial capitalism that has adversely affected both the Global South and marginalized populations in the Global North.

In this paper we analyze the 2022 formula-shortage-as-infant-feeding-crisis through the lens of the history of colonialism and critical theory in the anthropology of reproduction (Han and Tomori, 2022). We demonstrate, through our analysis of the 2022 infant feeding crisis, how the multinational CMF industry perpetuates racial capitalism and racialized health inequities and disparities through its operations as a neo-colonial enterprise. Although we focus on a case from one high-income country, multinational capitalism is a fulcrum of maternal and infant health inequalities between the Global North and South, while simultaneously perpetuating parallel health inequities within the US by race, gender, and class. Our paper offers a counter to analyses of infant feeding histories in the US that have overlooked these relationships.

First, we provide an overview of the colonial roots of the CMF industry in a global perspective. We then focus on how the mechanisms of exploitation remain entrenched in the US approach to infant feeding policies, regulation and investment, setting the stage for the current infant feeding crisis. In our discussion we highlight Abbott's activities because this company played a key role in triggering the crisis. However, we emphasize that the patterns of corporate behavior are not unique. Indeed, they demonstrate a pervasive pattern of undermining regulations and efforts to implement policies that support lactation. We conclude by providing policy recommendations for how to build a more resilient, equitable, explicitly decolonial infant feeding system that reclaims and restores deep cultural knowledge that has been systematically undermined in colonial systems.

## The CMF industry as a colonial system

The 2022 US CMF shortage is a crisis within a much broader global humanitarian maternal, infant, and young child malnutrition catastrophe anchored at the intersection of racism, colonialism, imperialism, and capitalism (Palmquist, 2022a). A local-global perspective is needed to contextualize the health inequalities perpetuated by this US CMF shortage, the critical policy gaps and structural inequities that produced it, and the global CMF industry's posturing to profit from it.

### Racial capitalism and the colonial roots of the CMF industry

Racial capitalism was first articulated by Robinson (2019) to describe the accumulation of wealth through racialized exploitation, which creates and sustains severe social inequities and racial oppression. As scholars elsewhere have demonstrated (Clarno, 2017), racial capitalism is the foundation of present-day globalized capitalist political economy. Massive profits have been generated by extracting wealth from the Global South and redistributing it throughout the Global North *via* routes and systems established during European colonization. Multinational and transnational corporations, including the CMF industry, have capitalized on these same systems to generate profits.

Many historical treatments of infant feeding history in the US have neglected to incorporate any substantive analysis of colonialism or racial capitalism. They also fall short in illustrating the ways that global and local economies and health inequities are connected. After all, the US has a history as a settler colony whose rise as a major global economic player was direct result of a particularly heinous form of racial capitalism, chattel slavery. As a framework to document the afterlives of European colonial regimes and global health inequalities, racial capitalism invites critical analysis of the contemporary global CMF industry as clearly deeply rooted in colonial systems of racialized oppression.

### Colonialism and it impacts

Around the turn of the 19th century, as European power and influence began to accelerate during the Industrial Revolution, the wealth gap between the Global North and the Global South became more pronounced (Wolf, 2010). At the same time, European colonial societies became increasingly stratified by gender, race, class, and geography. Colonial policies and practices that had displaced local subsistence-based economies or repurposed them to produce goods for national and international markets, set off a cascade of ecological degradation leading to population health inequities. In the wake of colonialism and with the rapid rise of global capitalism, Indigenous and local populations throughout the colonies became increasingly impoverished and sicker.

It is difficult to articulate the magnitude of mass death, destruction, and violence that European colonialism inflicted on indigenous populations around the world (Burton and Ballantyne, 2005; Tamale, 2020; Hokowhitu et al., 2021). The human, ecological, and planetary costs of enslavement include, but are not limited to, the intergenerational trauma and population health inequities resulting from centuries of slavery, genocide, forced displacement, family separation, environmental exploitation, and subjugation of Indigenous languages, religions, and cultural practices. Settler colonialism flourished by leaving racially oppressed populations traumatized, severely impoverished, and reliant on participation in market economies and global capitalism to survive.

Pregnant women, infants, and young children in the Global South were particularly harmed by the consequences of settler colonialism and racial capitalism. Throughout coastal and central countries of Africa in which there was severe social and economic upheaval due to the trans-Atlantic slave trade, control of female reproduction was central to colonial approaches to population control. In his analysis of the role that nutritional science played in the biopolitics of British colonialism in Africa, Nott (2021: p. 571–572) writes:

As a reaction to the gendered pressures of colonial government, protracted breastfeeding and sexual abstinence were increasingly untenable throughout the twentieth century, with birth spacing durations declining almost universally across the continent. This was in part, a result of colonial biopolitics. In the Belgian Congo, Africa's most extreme example of engineered pronatalism, “birth bonuses” worth “5 days” pay were given to contracted laborers working on plantations and mines across the colony. Even in less invasive spaces, such as the Gold Coast, state-sponsored baby shows formed a mechanism of “social regulation, if not social control” as early as the 1920s. Mothers were encouraged to bring up children according to western ideals and rewarded with sugar, soap and children's clothes when these conditions were met. The systematic influence of capitalist development compounded such policies. Again in the Gold Cost, male ownership of extra-subsistence produce severely undermined the value of childbearing, childrearing, and food production, the biologically and socially ascribed outputs of female labor. Accompanying the pervasive devaluation of such labor was a similarly pervasive pattern of gendered conflict.

Breastfeeding was disrupted by social and economic pressures introduced during colonial governance, which led to decreased birth spacing, early and abrupt weaning, increased maternal mortality, and increases in infant mortality and malnutrition.

### The rise of nutritional science, colonial medicine, and CMF marketing

Nutritional science and its integration into colonial medicine set the stage for commercial PIF to be introduced as a solution to nutritional deficiencies observed at the turn of the 20th century, during rapid economic and social transformations across Europe and its colonies (Wilhelm, 2020). Medical authority's increasing influence on infant feeding in Europe was instrumental to the Nestlé S.A. corporation in securing its reputation as a de facto humanitarian organization to assist colonial health in addressing infant malnutrition and related illnesses. From the mid 1920s, Nestlé was able to gain the trust of Swiss physicians by promoting and marketing their products directly to them and securing collaborations with them. Following World War II, Nestlé cultivated commercial and research partnerships with colonial doctors who were under increasing pressure to deal with food insecurity and hunger:

In 1956, for example, a medical representative from Nestlé embarked on a 6-week tour of the French African colonial territories, visited 112 doctors, sixty hospital services, fifty pharmacists, and organized forty clinical experiments…Similar programs took place in Southern Africa, British East Africa, and the Belgian colonies. Dozens of studies were conducted on Nestlé products across Africa in the 1950s (Wilhelm, 2019).

Medical professionals, NGOs, and the public increasingly viewed Nestlé CMF as a solution to infant malnutrition. These partnerships with colonial actors opened the door to a new market in which Nestlé could promote PIF throughout the African continent. Nestlé continued to diversify its marketing strategy, taking the promotion of its products out of clinical settings and into communities.

Among the most notorious practices was the use of salespeople dressed and acting as health care providers (“Nestlé nurses”), who were trained to deliver CMF marketing messages directly to pregnant and lactating women (Sasson, 2016). These messages, however, were not only informational; they were intentionally misleading and designed to cause women to stop breastfeeding and ostensibly coerce reliance on CMF. Nestlé provided free samples of formula to lactating women just long enough to disrupt lactation, most of whom were impoverished and living in severely economically depressed environments across the African continent, Asia, and Latin America. The conditions of formula feeding were catastrophic with some estimates placing the toll of Nestlé's marketing on both disruptions to breastfeeding and subsequent effects infant mortality in the millions (Jelliffe and Jelliffe, 1978; United States Senate, 1978; Sethi et al., 1994).

The “Nestlé controversy” and subsequent boycotts over its unethical and predatory marketing tactics have been extensively documented and analyzed (Sasson, 2016). Global outrage over the Nestlé corporation's economic and physiological exploitation for profit led to collective action for human rights policy known as the International Code of Marketing of Breastmilk Substitutes (the WHO Code), adopted in 1981 by the World Health Assembly (WHA) (Meier and Labbok, 2010). Importantly, however, while Nestlé's conduct is documented in the greatest detail, similar tactics along well-worn colonial paths were also used by other CMF companies (Manderson, 1982; Sasson, 2016). Moreover, increased awareness and transparency of the gender-based health disparities and inequities that are directly attributed to CMF marketing has done very little to stop the industry's unethical marketing practices (Baker et al., 2021a; World Health Organization, 2022). These marketing practices have consistently led to increased breastfeeding cessation and reliance on CMF (Baker et al., 2021a,b; World Health Organization, 2022), which disproportionately affect childbearing and lactating populations surviving the aftershocks of colonialism (Tomori et al., 2022a).

Of particular relevance for the present analysis is that Nestlé and other CMF companies effectively leveraged their position as biomedical authorities and humanitarian actors to create new marketing opportunities that would lead to reliance on their products and increase revenues globally. Aided by colonial assumptions of scientific superiority over Indigenous knowledge, the CMF industry successfully wove itself into the scientific apparatus that influences health care providers and nutrition policy and re-shaped default assumptions about infant feeding with CMF as the baseline. Furthermore, as formerly colonized nations across the world gained independence from colonial regimes, globalized market economies of CMF still allowed entities in the Global North to maintain control over reproduction from afar. Key to these efforts are aggressive tactics to shape trade law, food standards and marketing regulations that continue to provide opportunities to violate the WHO Code and undermine breastfeeding (Baker et al., 2021a; Russ et al., 2021; Tomori et al., 2022a).

### Racial capitalism and the impacts of CMF marketing in the US

As a former British colony, the effects of racial capitalism in the marketing of CMF perpetuated similar racialized health inequities in the US (DeVane-Johnson et al., 2018). Historically, enslaved African women were subjected to coerced separation from their newborns, to act as wet-nurse and caregiver to their enslavers' children, and to endure pressures to wean abruptly so that they may deliver more infants and contribute to the slave-dependent economy (West and Knight, 2017; Green et al., 2021). Upon emancipation, formerly enslaved African and African American childbearing people still were hired to breastfeed and care for white families' children. Once CMF was available in the US market, Black and brown families were identified as a market opportunity, in much the same way women in the Global South were (Wilhelm, 2019). Companies such as Abbott Nutrition turned their marketing efforts directly to Black women and women of color in economically depressed areas of the US (Seals Allers, 2017). The promise of healthier infants, liberation, and opportunities to engage in the US workforce for a wage persuaded many women to try CMF instead of breastfeeding. Instead of investing in policies and practices to ensure health equity through protection, promotion, and support of lactation and breastfeeding practices, the US has always chosen racial capitalism.

Policy makers and legislators at the highest levels of the U.S. government recognize that the U.S. economy depends on women in the workforce and generates enormous profit from international dairy exports and production of CMF. Together the government and the CMF industry mutually reinforce one another's wealth and power. They do this, in large part, by exerting strong economic and policy influences over the lactation and infant feeding practices of low and middle-income working class people, who due to structural racism in the US, are predominantly people of color (Seals Allers, 2017). The US has never endorsed the WHO Code or subsequent WHA Resolutions. The US has no federally mandated paid postpartum/family leave—a key structural determinant of breastfeeding (Tomori et al., 2022b). Racial and ethnic minority communities consistently have the worst breastfeeding outcomes and maternal and infant morbidity and mortality rates (Segura-Pérez et al., 2021; Centers for Disease Control and Prevention, 2022a).

In sum, CMF products were disseminated through international markets, many of which were established during western European colonial expansion throughout the Global South (Sasson, 2016; Wilhelm, 2020; Nott, 2021). The global expansion of the CMF industry has led to significantly lower breastfeeding rates, which is associated with substantial maternal and newborn morbidity and mortality incurred over the last 5 decades (Victora et al., 2016; Changing Markets Foundation, 2017; Walters et al., 2019; Baker et al., 2021a), particularly across the Global South and in socioeconomically disadvantaged and racially oppressed populations of the US and elsewhere in the Global North (Burnett et al., 2016; Tomori et al., 2022a). Historically, the CMF industry has acted both as a player in responding to infant feeding crises in international settings (Arvelo et al., 2010; Hipgrave et al., 2012; Hwang et al., 2021) as well as the cause of infant feeding crises. In both situations, the industry has generated hundreds of billions of dollars and comparable numbers of maternal and infant deaths (Changing Markets Foundation, 2017; Save the Children, 2018; Baker et al., 2021a).

## The contemporary US CMF industry and its disproportionate impacts: A neocolonial system

The current CMF shortage directly builds on and perpetuates colonial systems of exploitation. The formula industry has woven itself into governance and social life and continues to extract profits at considerable health costs (Walters et al., 2019; Baker et al., 2021a). The handful of multinational companies that govern this market hold enormous power in shaping infant feeding policy and options globally (Baker et al., 2021a). In the US, the industry's power is facilitated by a regulatory environment that favors corporate interests, profound inadequacy of social and health policies, and structural racism. The US is the only high income nation that lacks federal paid leave and universal health coverage (The Commonwealth Fund, 2021; Tomori, 2022). Additionally, the US lags behind other high income countries on metrics of health care affordability, outcomes, equity and efficiency (The Commonwealth Fund, 2021). This leads to millions of new mothers, disproportionately racialized minorities, who lack access to adequate care and have to return to work shortly after birth (Goodman et al., 2021). Formula companies create and maintain their market by actively exploiting these inequities and creating a largely captured market of families made to rely on their products through a variety of strategies, leaving them exceptionally vulnerable to this crisis.

### Market consolidation and the significance of the special supplemental nutrition program for women, infants, and children (WIC)

A key mechanism of how families become vulnerable in this infant feeding system is market consolidation, which is facilitated by trade laws that restrict competition. Only 4 companies control the US market, with Abbott accounting for about half of the market share, followed by Reckitt Benckiser Mead Johnson and Perrigo (31%), and Nestlé (8%) (Morris et al., 2022). Abbott's hold on the market is also driven by exclusive contracts for the WIC in 34 states and territories (Morris et al., 2022). Initiated in 1972 to serve the poor, early iterations of WIC undermined breastfeeding and incentivized CMF feeding. Indeed corporations were supportive of WIC because it provided them an opportunity to capture consumers and build brand loyalty (Kent, 2006). In the 1980s, a new, competitive bidding process was introduced by some states to reduce the costs of CMF for WIC. Companies lobbied against this change, but ultimately federal legislation in 1989 implemented the competitive bidding process across all states (Carlson et al., 2017). The CMF industry attempted to undermine the legislation by lowering discounts and coordinating among companies, which prompted an FTC investigation, lawsuits by 19 state attorneys general, and ultimately a settlement with the FTC in 1992 (Carlson et al., 2017). The competitive bidding process reduced the immediate profits to the CMF industry from WIC, however, access to WIC participants is sufficiently profitable for companies to continue their involvement (Carlson et al., 2017). Over the course of decades WIC has introduced progressively greater investment in breastfeeding promotion and support, but still invests much more heavily in purchasing CMF, constituting 42% of total WIC expenditure (Harris and Pomeranz, 2020).

Moreover, WIC's relationship with CMF companies continues to have additional unintended effects (Russ et al., 2021). Importantly, companies benefit from brand loyalty, the perceived endorsement of their product *via* WIC contracts, and recommendations of WIC brands by health care providers (Harris and Pomeranz, 2020). Oliveira et al. showed that a brand that was able to secure a new WIC contract could expand its market share by 74% through direct sales as well as due to results of these additional impacts on the perception of the brand (Oliveira et al., 2011). Additionally, Choi et al. (2020) demonstrated that winning WIC contracts had a strong impact on the profits, including in a substantial (42%) increase of toddler milks for the winning brand. WIC is the purchaser of over half of the formula consumed in the US, and Abbott provides half of the formula for about 2 million infants each month who are enrolled in WIC (Harris and Pomeranz, 2020; Morris et al., 2022). Abbott's Sturgis plant produces approximately one fifth of the formula consumed in the US (Berfield and Edney, 2022). With already existing periodic supply change challenges during COVID-19, the plant shut down set in motion the acute shortages we have seen throughout the spring of 2022 that disproportionately impacted WIC participants and other families with few resources.

### Capturing the market: Privatization and corporate influence on policies to intervene on exploitation

The few multi-national companies that control the US market engage in a wide range of corporate political activities both within and beyond the US that range from work through trade interest groups to political contributions. Internationally, lobbying efforts focus on a range of activities from limiting the influence of breastfeeding recommendations made by the WHO and WHA. A key target from its inception has been limiting efforts to implement the WHO Code, which was proposed in response to outcry about the harms of predatory marketing. For instance, lobbying efforts undermine regulatory standards for formula at the WTO so that countries cannot implement stricter regulations (Baker et al., 2021a).

In the US between 2007 and 2018, Abbott spent nearly 44 million USD on lobbying related to formula milks, constituting nearly 4/5th of the total by formula companies (Baker et al., 2021a). The companies lobbied the House, the Senate, the FDA, the State Department, Office of the US Trade Representative (USTR), and USDA. USDA lobbying is particularly notable since the FDA oversees WIC.

The above does not capture all the lobbying activities in the US, since companies also carry out lobbying *via* trade organizations. For instance, the International Formula Council, later renamed the Infant Formula Council (IFC), which includes all leading formula makers, lobbied against safety regulations for many years before the current crisis. In 2014, when the FDA proposed tightening rules, the trade group requested more time, questioned the evidence behind the prevalence of *Cronobacter* contamination, and the FDA relaxed the frequency of safety testing and provided exemptions for manufacturers. The IFC later petitioned even these rules, calling them burdensome and unnecessary (Fang, 2022). This set the stage for the current crisis.

### Evading safety regulations to maximize profits

The Sturgis plan has had a record of safety violations, including a recall of products in 2010 when beetle parts were found in formula, and inspections that revealed multiple other concerns (Morris et al., 2022). In 2019 some infants sickened after consuming Similac and Abbott found *Cronobacter* in some of its product, but no link was established to the Sturgis plant. However, a whistleblower report that was sent to the FDA and some members of Congress accused Abbott of a much deeper pattern of safety violations that entailed pressure from supervisors to cut corners to achieve production goals, systematic subversion of regulations despite known concerns, and falsification of records to mislead FDA regulators. It is not yet clear why this report was not investigated in a timely manner. An inspection in September 2021 revealed that Abbott had not maintained sanitary conditions at the plant, and that *Cronobacter* was also found in the prior year 2020 by Abbott. By this time there were additional reports of infants becoming ill with *Cronobacter*. After months of delay and more infants becoming ill, FDA inspectors returned to the plant in late January and found *Cronobacter* near production. The company's own records indicated that in February they found *Cronobacter* 20 times in the plant in a span of 2 weeks (Morris et al., 2022). These violations could have been avoided had the company invested in appropriate sanitary conditions and ensured the implementation of safety standards. But as the above shows, these investments were not made—instead they were overlooked in the interest of profits even as infants sickened and some ultimately died.

### Aggressive legal strategies

A recent New York Times investigation revealed that the CMF industry's history of circumventing safety regulations was coupled with aggressive legal strategies. Reporter David Enrich documents the history of cases of sick infants brought against Abbott and other CMF manufacturers that date back to at least 15 years (Enrich, 2022). Each of these cases were defeated and/or addressed with settlements that usually required non-disclosure agreements by powerful law firms hired by the industry. In one case, after a plaintiff declined a settlement, the law firm hired by Abbott mentioned their intention to introduce negative information about a different family member (a restraining order) that they claimed was linked to stress that might have caused the seizures of one child sickened by PIF to undermine trust in the family suing Abbott. This tactic succeeded even without mentioning that specific piece of evidence in court. After the trial the law firm succeeded in obtaining a court order to seal evidence and other trial materials “on the grounds that they contained confidential information about Abbott's testing and food safety protocols and ‘its sanitation, housekeeping and hygiene.”' Enrich (2022) Jones Day, the firm in this, and other cases highlighted in the article, has a history of similar tactics employed in corporate litigation across the industries they represent, which includes RJ Reynolds, known for its aggressive marketing of tobacco products, Purdue Pharma, manufacturer of oxycontin, which recently reached a historic settlement due to its role in the opioid crisis, and Smith & Wesson, a gun manufacturer, among others (Enrich, 2022).

### Corporate social responsibility and philanthropy

Other well-documented other efforts facilitate corporate consolidation of power. Corporate social responsibility campaigns and philanthropy, for instance, both operate to create a favorable image of these companies and provide a different avenue for expanding market share. Abbott, for instance, created the Abbott Fund in 1951, which claimed “to lead change and create new models for health care systems, improve nutrition and address other social needs” especially in child nutrition and other nutrition initiatives (Baker et al., 2021a).

Philanthropic involvement also participates in capturing health professionals. For instance, Abbott—along with Reckitt Mead Johnson and Nestlé, are funders of the AAP philanthropic efforts (American Academy of Pediatrics, 2022b). The AAP states that it “partners with companies and organizations whose support helps advance our mission for children.” The sponsorship comes with a disclaimer:

“A partnership does not imply endorsement of an organization's policies, products, or services and only begins after carefully reviewing factors such as corporate citizenship, shared values, and policy alignment.”

This statement presents these companies as aligned with the mission of improving child health, while the same companies actively engage in efforts to undermine regulations that would help achieve exactly this objective.

Emergencies constitute another opportunity for corporate philanthropy. Formula donations also help achieve a positive image even though in their actual effect they exceed need and undermine breastfeeding in situations where this places infants at much greater risks. Like other emergencies, COVID-19 similarly presented a marketing opportunity, with companies taking advantage of uncertainty about breastfeeding during the pandemic to offer health professional education and counseling about infant feeding (Baker et al., 2021a). Indeed, Abbott's net profits have more than doubled between 2019 and 2021 (from 3.6 to 7.1 billion USD) with significant gains in the US pediatric market (Perkins, 2022). In the wake of the current formula crisis, public health personnel reported that employees (marketing consultants) from companies who are industry competitors with Abbott Nutrition have approached hospitals with offers to distribute free formula and complementary gift bags in Baby Friendly hospitals (Palmquist, 2022b). Such distribution is not permitted under the WHO Code because of its well-documented role in undermining breastfeeding (Harris and Pomeranz, 2020). Emergencies are often approached as loopholes and opportunities for gaining more profit, and formula industries stand to profit off humanitarian crises, as they have always positioned themselves as the cause and the solution to breastfeeding emergencies.

### The role of health professionals and professional societies

Ostensibly benevolent efforts are part of the capture of scientists and health professionals, *via* infiltration of philanthropic efforts in professional societies, sponsorship of scientific meetings, conferences, and health care provider (HCP) training. Some of these tactics are manifest in the AAP philanthropy discussed above. Another well-known example is the Nestlé Nutrition Institute, which sponsors scientific meetings (Baker et al., 2021a). The Association of Women's Health, Obstetric and Neonatal Nurses (AWHONN) has a “strategic alliance” with Reckitt Mead Johnson (Association of Women's Health, Obstetric and Neonatal Nurses, 2022), and has previously partnered with Abbott Nutrition, which hosted industry symposia at the annual convention (Association of Women's Health, Obstetric and Neonatal Nurses, 2019). The home page for the strategic alliance explicitly mentions building loyalty with nurses:

“No other group of nursing professionals provides health care for more women and babies than AWHONN nurses. Let us help you create strong relationships and build loyalty [emphasis added] with these clinical leaders. Join the growing number of AWHONN partners who benefit from curated access to the full AWHONN nursing community. … AWHONN is your connection for strategic goals achieved with leading nurses caring for women and babies” (Association of Women's Health, Obstetric and Neonatal Nurses, 2022).

The level of access depends on the money paid. This kind of investment directly builds on the long-standing industry practice of using nurses in service of marketing efforts for CMF. As nurses have been ranked the most trusted profession in the US for 20 years (Gaines, 2022), an endorsement for products helps turn patients into customers. Importantly, this is not simply about providing patients with whatever infant feeding options they desire. Racial stereotypes play a role in early formula supplementation with Black mothers 9 times more likely to be given infant formula supplementation than their White counterparts (McKinney et al., 2016). Cultivating CMF industry relationships with HCP professional organizations *via* educational and “philanthropic” activities shapes HCP beliefs and behaviors (Piwoz and Huffman, 2015; Harris and Pomeranz, 2020) and directly feeds into this racist pipeline. Combined with pervasive racism in health care settings, including biases about Black women, inadequate breastfeeding support and other forms of discrimination, health care providers' favorable perception of CMF ultimately lead to disproportionate CMF supplementation (Gross et al., 2015; Thomas, 2018; Robinson et al., 2019). While the specific details of how the elements of corporate efforts to shape supplementation led to these racial inequities remain to be investigated, these efforts are similar to the role of colonial nutrition science in creating markets for the CMF industry among marginalized communities.

### Misleading families

In addition to the above, less visible components of the system, companies employ other well-established practices to build and retain their market share. Key aspects of this includes building relationships with baby clubs, sending product samples to pregnant women, providing infant feeding advice, and advertisements that make misleading scientific claims about how closely formula resembles breast milk, among others. Another element is to depict normal infant behaviors as problematic in need of intervention, often by using a product (Piwoz and Huffman, 2015; Vilar-Compte M, 2022). Health professionals play a key role in this, especially companies' efforts to cultivate specialty formulas, such as those for CMP allergies (van Tulleken, 2018). The relationship of demographically-targeted relationship-building and CMF use requires further attention.

A key element of these strategies is to capture and exploit the challenges and emotional vulnerabilities of new parenthood and infant care (Hastings et al., 2020). Abbott has been extremely successful in these efforts. Most notably their 2015 Sisterhood of Motherhood campaign picked up on the challenges of new motherhood and sought to build a new narrative. It depicted infant feeding decisions as highly moralized, and a matter of judgment that was overcome by uniting to rescue a baby. This pulled infant feeding decisions out of the domain of health and situated infant feeding as a matter of “lifestyle” (Hastings et al., 2020). It also helped to undermine any other framework by positioning these in the realm of moralizing—advocacy for breastfeeding would be seen as implying superior morality and a matter of individual decisions rather than the outcome of structural circumstances that are themselves shaped by industry interests (Tomori, 2014). The campaign was explicitly designed to counter diminishing market opportunities from dramatically increasing breastfeeding rates. It was the most successful campaign for Similac, increased sales and dramatically improved their media coverage (Hastings et al., 2020). Once again, these discourses perpetuate inequities as they overlook the structural barriers to breastfeeding, the health inequities that result from not being able to initiate or sustain breastfeeding, and corporations' own role in undermining breastfeeding.

### Shaping the narrative about infant feeding

In addition to advertisement, other media narratives also offer a powerful way of shaping public perceptions of corporations. The everyday behavior of corporations is rarely known to the public unless instances of severe misconduct come to light. Even in these cases, perceptions can be shaped and redirected. It is notable that after infants sickened and died, the conversation quickly shifted to the difficulties of breastfeeding. Debates on social media often devolved to individualized, morally loaded rhetoric (eg “just breastfeed”) and its counter. High profile news outlets featured multiple commentaries—mostly from women from privileged backgrounds—that focused on the challenges and costs of breastfeeding, often depicting breastfeeding as inherently flawed and requiring medical intervention (Tomori, 2014). In these pieces the immediate trigger of the CMF crisis—corporate misconduct—was no longer mentioned and the industry was depicted in a positive light as helping to save infants who would otherwise starve (Hausman, 2003; Tomori, 2014). Even more thorough investigations of corporate misconduct fail to engage with the full scope of corporate activities that facilitate expanding markets—such as the activities above that systematically undermine breastfeeding.

Moreover, in most media depictions there is little recognition that structural conditions could be altered to support breastfeeding and make it far less challenging, or how infant feeding systems could be built that are less reliant on CMF. Decades of literature from anthropology, sociology and related disciplines demonstrate that moralized narratives of infant feeding are harmful and conceal structural failures (Tomori, 2014; Tomori et al., 2018). Indeed, we have ample evidence, including from the US, that systemic changes can strongly influence breastfeeding prevalence, which has significant public health implications (Rollins et al., 2016; Tomori et al., 2022b). Whether breastfeeding “works” or not is dependent on its context. Media outlets actively shape perceptions by featuring pieces that generate controversy (clicks), and media ownership has been strongly linked to corporate interests (McKee and Stuckler, 2018). Unwitting commentators can be easily swept up in efforts to present an individual choice narrative about infant feeding that ultimately disproportionately impacts Black, Indigenous, and Latine women.

### Profits over safety: Stock buybacks and dividends

The prioritization of profits over safety has paid off for Abbott. The company was able to spend on stock buybacks and dividends. In December it authorized 5 billion dollars in stock buybacks to boost its share price, which increased dividends by 25% (Perkins, 2022). Recall that about half of these sales rely on WIC participants, who are poor and where racial and ethnic minorities are disproportionately represented. These practices are now under scrutiny and subject of Congressional hearings. However, company stock prices have already gained back much of what was lost after the initial press coverage that linked the formula shortage to unsanitary conditions at Abbott's Sturgis plant.

### Disproportionate impacts of a neocolonial system

All of the above corporate activities demonstrate how WIC participants were especially exposed to the impacts of the shutdown of the Sturgis plant. WIC participants were more negatively impacted by the formula shortage because they were reliant on Abbott products, which were precisely the ones out of stock. Importantly, WIC participants disproportionately rely on formula because of the structural barriers to breastfeeding which affects them most. WIC eligibility is determined by income, and poor people, disproportionately racialized minorities, are also those who are least likely to be able to have access to paid postpartum leave, to hold employment that provides a supportive environment for lactation, and to have access to skilled lactation support.

Although WIC's breastfeeding programs, which include peer counseling and an enhanced package that supports the nutritional needs to lactating parents, have mitigated some of these structural barriers, they are insufficient in an infant feeding landscape steeped in racism and structural inequities. Together with the colonial roots of the industry's unethical marketing practices, the historical involvement of formula companies in WIC, the continued disproportionate impacts of marketing on poor, racialized minorities, the current crisis demonstrates the impacts of a neocolonial system of wealth extraction. This system makes marginalized people reliant on an industry that creates a narrative of benevolence, when in fact it undermines their health at every step and exposes them to the consequences of their unethical practices. The system in the US is clearly part of a broader global system of neocolonial wealth extraction that drives profits derived from poorer people and minorities to the pockets of wealthy executives and shareholders in the Global North.

## Policy recommendations to build a decolonial system of infant feeding

A starting point for building an effective system of infant feeding begins with structural changes that address the policy and regulatory environment that has led to contemporary failures. The shortage and its disproportionate impacts represent just the tip of the iceberg of the consolidation of corporate power of the global infant formula industry in the hands of a few multinational companies. As we face an increasing set of emergencies, from the current COVID pandemic to new pandemics and climate change (Swinburn et al., 2019), similar impacts will become more frequent even in relatively wealthy settings but always with disproportionate, specific impacts within these settings. In poorer settings, the devastation they can cause will be on an even greater scale.

### Removing corporate influence on the policy environment

The foundations of change at the policy level must start with a thorough investigation and restrict corporate lobbying and other efforts that use money and influence to shape global and local infant feeding policies. The implementation of the WHO Code and subsequent WHA resolutions is clearly necessary to address predatory marketing. Another key element of the WHA resolutions is HCP education and skilled lactation support without industry influence. Health professional and scientific societies must dissolve their partnerships with the formula industry to achieve this aim. However, addressing the influence of multinationals on politics and policy must go further than the Code. This is a particular challenge in the US that has increasingly enabled corporate capture of politics, and US corporate interests also play a disproportionate role on the global stage (Baker et al., 2021a).

Enhanced safety regulations and independent oversight of their implementation must be imposed based on scientific evidence free of conflicts of interest. Corporations like Abbott and others and their trade groups cannot be involved in shaping these regulations and cannot be allowed to self-regulate.

### Greater investment in structural support for breastfeeding

The removal of corporate influence must be paired with greater investment in structural supports for lactation. In a recent review we found that few interventions focused on the policy environment despite strong evidence that upstream structural factors play a significant role in shaping health outcomes (Tomori et al., 2022b). For US parents probably the greatest barrier to breastfeeding remains the lack of paid leave. This is an issue that cannot be left unaddressed if the US is to build a more equitable infant feeding system. Millions of US mothers currently breastfeed not because of supportive policies but by sheer determination to overcome the odds. Indeed, this determination and persistence is seen in breastfeeding prevalence that is higher than in the UK where there is parental leave and access to universal health care.

The US should enact policies that subsidize and incentivize the universal implementation of the Baby Friendly Hospital Initiative (BFHI) and expand access to skilled lactation support both within health systems and in the community (Asiodu et al., 2021; Tomori et al., 2022b). This requires investing more resources in lactation training and education by the government and non-profits that are not associated with industry interests (Waterston and Yilmaz, 2016; Grummer-Strawn et al., 2019).

WIC remains a crucial resource for more equitable access to breastfeeding education, counseling, and skilled peer support among economically disadvantaged families, who are predominantly families of color. The structure of the WIC program and its proportion of investment in breastfeeding must be re-examined. Significant scale-up of investment in maternal nutrition and breastfeeding may increase program costs on one end, but has the potential to enable more participants to attain their breastfeeding goals without negative unintended consequences. Similarly, national policy reform should specifically address the formula purchasing model of WIC to minimize endorsement of specific products and positioning WIC as an unwitting marketing arm of the CMF industry.

While pasteurized donor human milk has been included in recommendations for responding to the formula shortage (Academy of Breastfeeding Medicine, 2022), we have yet to see substantial investment to scale up non-profit milk banking in the US to meet the increased needs. Instead, nearly all the resources poured into the response has focused on remediation and expansion of the CMF industry. Non-profit milk banks play a pivotal role in strengthening a public health infrastructure that is supportive of breastfeeding. A robust network of non-profit milk banks also has potential to improve the resilience of local and national health systems in public health emergencies, like COVID-19 and protracted formula recalls or shortages (Israel-Ballard et al., 2019).

In Colorado, Governor Polis has signed an agreement to support milk banks in response to the formula shortage (Governor of Colorado, 2022). Many milk banks that are part of the Human Milk Banking Association of North America (HMBANA) have sponsored milk donation drives to meet the increased demands among medically fragile hospitalized infants during the formula shortage. Some have also been able to make donor milk available for limited community distribution. While these efforts are responsive to the formula shortage emergency, long-term, structural changes are required to foster a not-for-profit milk banking system that is equitable and provides sustained access to quality, lifesaving donor human milk.

A model for this has been implemented in Brazil, and it has been highly successful (Pires, 2014). In India where maternal-infant morbidity and mortality is among highest in the world, governmental ownership and investment has been central to planning the country-level scale up of milk banking (DeMarchis et al., 2017). This commitment to ensuring that the 30%-50% of newborns admitted to neonatal critical care, and 10–20% of term newborns in need of supplemental feedings, have equitable access to pasteurized donor human milk may reduce the country's reliance on CMF and preventable infant malnutrition, stunting, and wasting. Integration of human milk banking into national public health infrastructure, as part of a holistic bundle of care services for mothers and newborns, would lead to greater breastfeeding equity and positive population-level outcomes on breastfeeding (DeMarchis et al., 2017). A key challenge is to enact legislation that would ensure such a scale-up in an equity-centered manner so that milk banking does not become subject to monopolistic forces or become captured by commercial interests (Miracle et al., 2011; Thibeau and Ginsberg, 2018; Hartmann, 2019; Fang et al., 2021). Protection of non-profit models for human milk banking is needed in the U.S., as commercial entities are increasingly posturing to shape policies, legislation, and market monopolies, under the guise of donor milk safety, using similar tactics as the CMF industry. Increased commercialization of human milk, whether through for-profit milk banking or human milk-derived products, poses critical threats to health equity and would disproportionately disenfranchise racially marginalized and economically-disadvantaged communities. Importantly, the US currently lacks a federal infant and young child feeding in emergencies (IYCF-E) policy or related training and implementation infrastructure to ensure first-food security in the wake of disasters and emergencies. It lacks a system to ensure coordination of personnel, supplies, and support for infants who are breastfed or fed with expressed human milk or to ensure that the timely procurement and delivery of all the supplies needed to meet the needs of formula reliant infants. A robust and comprehensive plan is essential to prepare and respond to multiple intersecting threats such as the concurrent COVID-19 pandemic and wildfires from climate change, for instance. This plan must explicitly lay out how infant feeding inequities rooted in structural racism are addressed, so that inequities are not further deepened by these emergencies (United States Breastfeeding Committee, 2022a). Gaps in U.S. IYCF-E policies and programs have been only partially addressed through the new HHS Maternal-Child Emergency Planning toolkit and an IYCF-E Toolkit released just this year by the CDC (Centers for Disease Control and Prevention, 2022b; United States Department of Health Human Services, 2022). A first step in policy reform for IYCF-E is to follow WHO guidance that incorporates human rights principles and prioritizes vulnerable populations and those who are most marginalized (Meier and Labbok, 2010; Tomori et al., 2022c).

### Reclaiming Indigenous systems of knowledge, decolonizing policymaking

In building a new infant feeding system, concerns of those most affected by the neocolonial systems of exploitation must be centered and should be included throughout the decision making and policy process. This requires that Black, Indigenous, Asian, and Latine community members are not only invited to participate, but that the process explicitly addresses the violent disruption of families, communities, and entire lifeways through colonialism and recognizes ongoing efforts of leaders from marginalized communities to restore and reclaim deep cultural knowledge. The policy process must have an equity-oriented, decolonial framework (Tuhiwai-Smith, 2021) that aligns with community goals and ensures that resource allocation is consistent with these efforts. Numerous, community-driven efforts are underway to achieve these goals. For instance, Indigenous lactation experts, Camie Goldhammer (Sisseton-Wahpeton) and Kimberly Moore-Salas (Navajo), have developed the Indigenous Breastfeeding Counselor program and have carried our trainings across the United States and Canada (Nowell, 2017). Rhonda Lee Grantham, an Indigenous Midwife and Herbalist from the Cowlitz Nation, founded the Center for Indigenous Midwifery in the State of Washington (Grantham, 2022). Similar efforts have been initiated and gaining support across the country as well as in Canada (First Nations Development Institute, 2022). For instance, Indigenous midwife and lactation expert, Stephanie George and colleagues serve their First Nations community at the Six Nations Health Services (Six Nations Health Services, 2022). The M.A.N.A. Pasefika doula program in San Francisco, California, supports people of Pacific Islanders of Melanesian, Micronesian, and Polynesian ancestry (M.A.N.A. Pasefika, 2022). An Asian and Pacific Islander Breastfeeding TaskForce was established in 2017, and received acknowledgment from the California Breastfeeding Coalition in removing barriers to breastfeeding in Asian and Native Hawaiian and Pacific Islander communities (Tseng, 2021). A large number of organizations support Latine breastfeeding and lactation across the country (United States Breastfeeding Committee, 2022b). ASI (Alimentacion Segura Infantil) in Puerto Rico specializes in supporting IYCF-E in Puerto Rico (ASI, 2022), which recently experienced catastrophic flooding due to Hurricane Fiona (Taylor, 2022). Reaching our Sisters Everywhere (ROSE), HealthConnect One, and the Black Mothers Breastfeeding Association (BMBFA) are leading work that actively centers the needs of Black communities (Asiodu et al., 2021) (68). ROSE held its 11^th^ Summit, “1619-2022 Black Breastfeeding & Birth Justice Summit” (ROSE, 2022) and has previously developed an African American Breastfeeding Blueprint addressing racial breastfeeding inequities and how to overcome them modeled on the 2011 US Breastfeeding Blueprint (ROSE, 2019). The Birth, Lactation, Accommodation, Culture, Kinship (B.L.A.C.K.) course, focusing on aspiring Black lactation professionals, was launched in 2021 (Walker-Tibbs et al., 2022). In the summer of 2022, during National Breastfeeding Month, we celebrated Indigenous Milk Medicine Week (formerly Native Breastfeeding Week, in its third year), Asian American Native Hawaiian and Pacific Islander Breastfeeding Week (in its second year), and Black Breastfeeding Week (in its tenth year) (United States Breastfeeding Committee, 2022c). A number of states have formally adopted resolutions recognizing these events (see e.g. Governor of Michigan, 2022). Each of these events and documents explicitly addresses the colonial, oppressive and violent roots of disruptions in breastfeeding and demonstrates the reclaiming, restoring and strengthening of traditions. The US Breastfeeding Committee actively represents these interests and advocates for equity-oriented policies.

## Conclusion

In this analysis we have used the current US formula shortage to highlight the colonial roots of the contemporary formula industry that is dominated by just a handful of multinational companies. We have argued that the unequal, racialized impacts of the current formula shortage were predetermined by the structure and practices of the industry that perpetuates a system of neocolonial wealth extraction, making those most marginalized reliant on their products and drawing from them to generate profits at significant economic and health costs. We explored how this system is sustained by corporate influence that shapes the entire policy and regulatory environment as well as media discourse and public opinions. Finally, we have made recommendations for policy changes that could be undertaken to limit the influence of these multinational corporations and to build a more equitable, decolonial infant feeding system. While these policy actions may seem daunting, the damage caused by multinational capitalism on infants, families, and communities in the US and globally is profound and ongoing. As of late September 2022, the formula crisis has eased but stocks have not returned to pre-crisis levels and investigations are ongoing in tracing the specific failures that led to the acute crisis (Jones, 2022; Rosenberg, 2022). To address root causes, however, a much deeper reckoning is necessary. Each step taken to limit corporate influence and cultivate an alternative system will cultivate benefits beyond the present crisis—across generations.

## Author contributions

CT conceived the paper with AP's input. Both authors participated in developing the policy analysis, reviewing the literature, and writing the manuscript. Both authors contributed to manuscript revision, read, and approved the submitted version.

## Conflict of interest

The authors declare that the research was conducted in the absence of any commercial or financial relationships that could be construed as a potential conflict of interest.

## Publisher's note

All claims expressed in this article are solely those of the authors and do not necessarily represent those of their affiliated organizations, or those of the publisher, the editors and the reviewers. Any product that may be evaluated in this article, or claim that may be made by its manufacturer, is not guaranteed or endorsed by the publisher.
